# School-based intervention to address self-regulation and executive functioning in children attending primary schools in remote Australian Aboriginal communities

**DOI:** 10.1371/journal.pone.0234895

**Published:** 2020-06-24

**Authors:** Bree Wagner, Jane Latimer, Emma Adams, Heather Carmichael Olson, Martyn Symons, Trevor G. Mazzucchelli, Tracy Jirikowic, Rochelle Watkins, Donna Cross, Jonathan Carapetis, John Boulton, Edie Wright, Tracy McRae, Maureen Carter, James P. Fitzpatrick

**Affiliations:** 1 Alcohol and Pregnancy and Fetal Alcohol Spectrum Disorder Research Team, Telethon Kids Institute, The University of Western Australia, Perth, Western Australia, Australia; 2 Sydney School of Public Health, Faculty of Medicine and Health, The University of Sydney, Sydney, New South Wales, Australia; 3 Seattle Children’s Research Institute, Seattle, Washington, United States of America; 4 University of Washington School of Medicine, Seattle, Washington, United States of America; 5 National Health and Medical Research Council FASD Research Australia Centre of Research Excellence, Perth, Western Australia, Australia; 6 Telethon Kids Institute, The University of Western Australia, Perth, Western Australia, Australia; 7 Child and Family Research Group, School of Psychology, Curtin University, Perth, Western Australia, Australia; 8 Brain, Behaviour and Mental Health Research Group, School of Psychology, Curtin University, Perth, Western Australia, Australia; 9 Division of Occupational Therapy, Department of Rehabilitation Medicine, University of Washington School of Medicine, Seattle, Washington, United States of America; 10 Health Promotion and Education Research Team, Telethon Kids Institute, The University of Western Australia, Perth, Western Australia, Australia; 11 CoLab–Collaborate for Kids, Telethon Kids Institute, The University of Western Australia, Perth, Western Australia, Australia; 12 Perth Children’s Hospital, Perth, Western Australia, Australia; 13 The University of Newcastle, Newcastle, New South Wales, Australia; 14 Western Australian Department of Education Kimberley Education Region, Broome, Western Australia, Australia; 15 Nindilingarri Cultural Health Services, Fitzroy Crossing, Western Australia, Australia; University of Wollongong, AUSTRALIA

## Abstract

Executive functioning and self-regulation influence a range of outcomes across the life course including physical and mental health, educational success, and employment. Children prenatally exposed to alcohol or early life trauma (ELT) are at higher risk of impairment of these skills and may require intervention to address self-regulation deficits. Researchers partnered with the local Aboriginal health organization and schools to develop and pilot a manualized version of the Alert Program® in the Fitzroy Valley, north Western Australia, a region with documented high rates of fetal alcohol spectrum disorder and ELT. This self-controlled cluster randomized trial evaluated the effect of an 8-week Alert Program® intervention on children’s executive functioning and self-regulation skills. Following parent or caregiver consent (referred to hereafter as parent), 271 students were enrolled in the study. This reflects a 75% participation rate and indicates the strong community support that exists for the study. Teachers from 26 primary school classrooms across eight Fitzroy Valley schools received training to deliver eight, one-hour Alert Program® lessons over eight-weeks to students. Student outcomes were measured by parent and teacher ratings of children’s behavioral, emotional, and cognitive regulation. The mean number of lessons attended by children was 4.2. Although no significant improvements to children’s executive functioning skills or behavior were detected via the teacher-rated measures as hypothesized, statistically significant improvements were noted on parent-rated measures of executive functioning and behavior. The effectiveness of future self-regulation programs may be enhanced through multimodal delivery through home, school and community based settings to maximize children’s exposure to the intervention. Despite mixed findings of effect, this study was an important first step in adapting and evaluating the Alert Program® for use in remote Australian Aboriginal community schools, where access to self-regulation interventions is limited.

## Introduction

The ability to regulate emotion, behaviour, and thought relies on a set of integrated brain processes known as the executive functions. These top-down (deliberate) cognitive functions are commonly believed to include inhibition, working memory, and cognitive flexibility [1, 2]. Additional higher-order executive functions are thought to include planning and reasoning [1]. Working together, these cognitive processes support individuals to purposely solve problems and function adaptively throughout life [3]. Self-regulation is a related concept [4, 5], referring to the volitional ability of a person to modify or maintain arousal states [6], emotions, thoughts, or behaviors appropriate to a situation [7]. Although research to define the exact association between executive functioning and self-regulation continues [7], a bidirectional relationship is thought to exist [8]. Firstly, the executive functions underlie self-regulation [9], meaning an individual employs top-down cognitive processes to gain control over their emotions, behaviors, and thoughts [7, 10]. Secondly, bottom-up self-regulatory processes enable top-down executive functioning. That is, the body receives and processes sensory information to modulate arousal, emotion, and attention, which impacts executive functioning [7, 8]. Ultimately, executive functioning and self-regulation influence a range of outcomes across the life course, including mental and physical health, educational success, and employment [11–13].

The nature of schooling demands constant use of children’s executive functioning and self-regulatory abilities [14]. For example, students must follow instructions, apply classroom rules, solve maths problems, comprehend texts, get along with peers and teachers, remember equipment, and transfer knowledge between situations. Without well-developed executive functioning and self-regulation skills, students can struggle with these basic school tasks. In these instances, teachers may view students as having problematic or disruptive behavior [15, 16]. Additionally, impairment in executive functioning and self-regulation amongst students can lead to disengagement from school, student-teacher conflict, poor academic achievement, and disrupted learning for other students [15, 17]. Deficits in executive functioning and self-regulation are widely reported as ‘hallmarks’ of fetal alcohol spectrum disorder (FASD) [18–20], a pervasive neurodevelopmental disorder arising from prenatal alcohol exposure [21]. Children’s exposure to adverse childhood experiences (ACEs), including stress and poverty, can also impair executive functioning and self-regulation development [22]. ACEs are sometimes referred to as early life trauma (ELT) [23]. Given evidence of the malleability of the executive functions and self-regulation [9], a key opportunity exists to develop these skills amongst children with or at risk of dysfunction.

Aboriginal leaders from the remote Fitzroy Valley region of Western Australia (WA) have been concerned about the potential of FASD and exposure to ELT to impact the behaviours of children in their local community. In 2009, local leaders developed a multifaceted strategy called the *Marulu FASD/ELT Strategy* (Marulu means ‘precious, worth nurturing’ in the Bunuba language) [24]. This strategy prioritized three aims: FASD prevention and diagnosis, and appropriate support for children and families affected by FASD and ELT. As part of this strategy, Fitzroy Valley leaders partnered with researchers to conduct Australia’s first FASD prevalence study otherwise known as the *Lililwan Study* (Lililwan means ‘all the little ones’ in Kimberley Kriol) [25]. In this study, 108 Fitzroy Valley children born between 2002 and 2003 were assessed. This study found a FASD prevalence of 194 per 1,000 school-aged children [26], this rate is considered extremely high. In contrast, global FASD prevalence has been reported as 7.7 per 1000 children [27]. Interestingly, of all the children assessed in this study, 30% were identified as having executive functioning impairment (albeit through brief assessment) and 26% as having behavioral problems [26, 28]. Preliminary Lililwan Study data also indicated 90% of children had been exposed to ELT with over 50% of children experiencing greater than three different ELTs [26]. There are significant stressors for Fitzroy Valley children and families arising from poverty, family violence, limited access to fresh food and transport, overcrowded and insecure housing, chronic illness, and separation from parents [25]. The 2005 Western Australian Aboriginal Child Health Survey also reported that 22% of carers of children living in the region had experienced seven or greater ‘life stress events’ within their family [29]. Therefore, in addition to the high prevalence of FASD, exposure to ELT may have contributed to the high rates of executive functioning and behavioral problems identified amongst children in the Lililwan Study [26].

As a result, community members, including school staff, requested training and support to address disruptive student behavior as a means to improve children’s learning [25]. However, the geographical isolation of Fitzroy Valley communities meant that available pediatric therapies were limited. To overcome service barriers, researchers, Fitzroy Valley communities, and schools together identified the Alert Program® as a feasible and appropriate program to improve students’ executive functioning and self-regulation skills [30].

The Alert Program®, developed by occupational therapists, teaches children about self-regulation through the analogy of a car engine. Children are taught that ‘just like a car engine, our bodies can be in a high, low or just right speed’ [6]. Through this analogy, children learn to recognize their own arousal states before learning to use strategies and tools from five sensorimotor categories (mouth, body, touch, look, listen) to help change or maintain an optimal level of arousal (engine/alertness levels) for different situations [6]. Upskilling of adults in key Alert Program® concepts is also a core aspect of the program [31].

The Alert Program® has previously been adapted and trialled in schools [32–34] and FASD clinic settings [35–38], with some reported improvements to children’s executive functioning and self-regulation abilities on a range of standardized and non-standardized measures [32, 34, 35, 37–39]. However, until this research project, no Australian Alert Program® studies, or studies with predominantly Indigenous participants, have been reported [40]. Therefore, this study aimed to assess the efficacy of an 8-week Alert Program® intervention, delivered by classroom teachers to children attending primary school in remote Australian Aboriginal settings, on students’ executive functioning and self-regulation skills. Student outcomes were measured by teacher and parent ratings of their behavioral, emotional, and cognitive regulation.

## Methods and materials

A detailed protocol for this study was prospectively published and registered with the Australian New Zealand Clinical Trials Registry (ACTRN12615000733572) [41]. A detailed description of the development and content of the intervention training and delivery has also been reported elsewhere [30, 41, 42].

### Ethics approvals

Ethics approvals were provided by The University of WA (RA/4/1/7234), the WA Aboriginal Health Ethics Committee (601), and the WA Country Health Service (2015:04). The study was also approved by the WA Department of Education and the Kimberley Aboriginal Health Planning Forum Research Sub-Committee.

### Community collaboration

This study addressed a community identified need to support the development of adaptive student behaviors in the Fitzroy Valley schools. Prior to this study, researchers undertook a year-long formative process in partnership with local leaders (MC, EW), stakeholders, families, schools, and Aboriginal community researchers to develop culturally secure and contextually feasible research processes and protocols [30]. During a subsequent pilot study, researchers gathered feedback from school staff on the suitability of training, lessons, and resources for the remote school context [30, 42]. Guided by this feedback, researchers refined the intervention in consultation with the original Alert Program® authors. Results of the formative stage of this project and results of the pilot study have been published elsewhere [30, 42].

### Community researchers

Recognising the cultural diversity of the region, 18 Aboriginal community members from the four main Fitzroy Valley language groups were employed to provide essential expertise in relationship brokerage, cultural protocols, language translation, and general research assistance as community researchers [43, 44]. The two-way partnerships fostered between Aboriginal and non-Aboriginal research staff were vital for ensuring cultural safety for participants and researchers alike [45].

### Setting

The study took place at four government and four independent community schools in the remote Fitzroy Valley. This culturally diverse region is home to approximately 3,500 mainly Australian Aboriginal people, from the Bunuba, Walmajarri, Gooniyandi, and Wangkatjungka language groups [46]. Kimberley Kriol and local Aboriginal languages are commonly spoken, with English an additional language for many. Residents live across 45 distinct Aboriginal communities spread over an area of 40,000 square kilometres [47]. The remoteness of schools and communities means access to facilities such as grocery stores, health services, recreation facilities, and social services is limited. Each day, children may travel up to 100 kilometres one way from their home community to attend their closest school.

### Study design

This study was a self-controlled cluster randomized trial in which primary school classrooms in each cluster of schools received the intervention at one of four time-points (school terms two or three, 2016 or 2017).

### Randomization and cluster assignment

Schools were assigned by researchers to one of four clusters based on geographic proximity and school size. This responded to the challenge researchers experienced travelling vast distances to collect data from participants in different schools within the same cluster at varying calendar times. Pre-assigning schools to clusters also ensured a relatively even distribution of participants across each cluster, given that school size varied from six to 110 students. Clusters included one to three schools. Randomization to the intervention schedule occurred at the cluster level as determined by a computer-generated randomization pattern.

### Inclusion criteria

All students enrolled in grades (years) one to six (5.5–12.5 years of age) at any of the eight study schools at the time of recruitment in their cluster were eligible to participate in the study with written parent consent. All teaching staff with responsibility for delivering the Alert Program® were also eligible to participate in the study with informed written consent.

### Sample size

The available study population was 363 students [48, 49]. Community leaders requested that all eligible Fitzroy Valley families be invited into the study. Given the non-standard study design, sample size estimates were calculated using standard methods for a cross-over randomized control trial with 60 participants per cluster across time-points with 80% power to detect an effect at the 0.05 significance level [41].

### Recruitment and consent

Initial approval to conduct the study was provided by community Elders and school principals following a series of engagement visits to the Fitzroy Valley [30]. Research assistants and Aboriginal community researchers then visited families together to explain the purpose of the study and to seek written consent for their child(ren)’s involvement. Community researchers provided verbal translation of study materials into Kimberley Kriol or other Aboriginal languages when required. Separate consent items included: (1) collection of student outcome data; (2) access to students’ medical records and students’ school attendance records; and (3) permission to take photos and videos of students during Alert Program® lessons. Researchers obtained informed written consent from teachers following a school staff information session. For this study, teachers consented to completing teacher-rated questionnaires for participating students and to record lesson information and student attendance data.

### Alert Program® intervention

While a detailed description of the intervention has been described elsewhere, briefly, all school staff, including Aboriginal and Torres Strait Islander education officers (AIEOs), were invited to attend two one-day training sessions facilitated by the study coordinator [30]. It was important that AIEOs attended the training as they work in two-way teams with classrooms teachers to support teaching and learning activities including the translation of instructions or concepts from English to Kimberley Kriol for students when required. Training session one occurred before teachers delivered the first Alert Program® lesson, and training session two occurred between weeks three and four of program delivery. The training covered the history and purpose of study, and general information about FASD and executive functioning [30]. Staff also viewed the Alert Program® Online Course modules one to five, and had the opportunity to ask questions during the two sessions. The Alert Program® Leader’s Guide, lesson manual, equipment boxes, and other resources (such as picture cards and posters) required for lesson delivery were provided to teachers, who were encouraged to contact the study coordinator if they had further queries pertaining to lesson delivery or use of resources.

Classroom teachers were asked to deliver a one-hour manualized Alert Program® lesson to all students in their class, once a week, for eight consecutive weeks (8 hours in total). The intervention was designed by adapting Stage One and Two of the original three stage Alert Program®. Stage Three relates to independent and ongoing use of sensorimotor strategies by participants [6]. The resource intensive nature of this study, given participating schools were widely spread, meant that the longer-term follow-up necessary for evaluating the effectiveness of Stage Three was not possible. Therefore it was decided to deliver this adapted version of the Alert Program®. The intervention dose used in this study reflects the dose delivered in other studies evaluating the effectiveness of the Alert Program® in schools, and therefore was considered a justifiable starting point [32, 34]. During lessons one to three, students developed an awareness and feel for arousal levels in the body and practiced using the engine language. During lessons four to eight, students experimented with strategies from the five sensorimotor categories to change or maintain their arousal levels (engine/alertness levels) [6]. Teachers were also encouraged to embed program language and strategies throughout the school day [30].

### Outcome measures

#### Sutter-Eyberg Student Behavior Inventory-Revised (SESBI) and Eyberg Child Behavior Inventory (ECBI)

The 38-item SESBI is a teacher-rated measure of student disruptive behavior at school, while the 36-item ECBI is a similar measure of child behavior at home [50]. Both questionnaires were used as proxy measures of problems in self-regulation. The SESBI and ECBI include two scales: the Intensity Scale, which measures frequency of disruptive behaviors on a scale from 1 ‘never’ to 7 ‘always’; and the Problem Scale, in which respondents rate each behavior as being a problem, 0 ‘no’ or 1 ‘yes’. A reduction in scores on a scale indicates improvement. Both the SESBI Intensity and Problem scales have high internal consistency (α = .98, α = .96) and high test-retest coefficients (*r* = .87, *r* = .93) [50]. Internal consistency and test-retest coefficients for the ECBI Intensity and Problem scales are also high (α = .95, α = .93; *r* = .80, *r* = .85) [50]. The SESBI Intensity Scale was chosen as the primary outcome for the current study.

#### Behavior Rating Inventory of Executive Function 2 Teacher and Parent Screening Forms (BRIEF2-SF)

The 12-item teacher-rated and parent-rated BRIEF2-SF comprise overall measures of global executive function that include cognitive, behavioral, and emotional regulation items [51]. Items are rated as occurring 1 ‘never’, 2 ‘sometimes’, or 3 ‘often’ and yield a score indexing the overall level of executive functioning. Lower scores indicate fewer executive functioning difficulties. Both the teacher and parent versions of the BRIEF2-SF have high internal consistency (*α* = .91, .89), test-retest reliability (*r* = .87, .79), and correlate strongly with the full BRIEF2 Global Executive Composite Score (*r* = .95, .96) [51]. Parents were not asked two of the cognitive regulation items from the BRIEF2-SF (Q6 and Q7), as pilot testing deemed these questions irrelevant to parents’ experiences in these remote communities [30]. Instead, excluded questions were assigned a score determined by pro-rating the remaining three cognitive regulation items, as suggested by the BRIEF2-SF developers (P. Isquith, personal communication, October 14, 2015).

In the context of the current study, the BRIEF2-SF was used as a secondary outcome measure. The screening form was chosen because of the battery of measures being used, and the need to reduce the burden for respondents by using a screening form measure. This was considered acceptable given that while the level of detail about specific profiles of executive function is limited in the screening form, it still provides a sound estimate of global executive function and is strongly correlated with the full BRIEF2 Global Executive Composite Score [51].

### Data collection

Data were collected over two-week periods per the timeline in Table 1 [41]. For the control condition, data were collected for clusters two, three and four, and matched the timing of the preceding intervention cluster. Cluster one did not have a control period for several reasons: data collection was not feasible in school term one (February–March) due to cultural and school business, and the likelihood of seasonal flooding causing road closures. Also, given this was the beginning of the school year, teachers may have had insufficient knowledge of the student prior to be able to accurately complete questionnaires. For the intervention condition, data were collected immediately before (pre) and after the intervention (post), and at eight to nine-weeks follow-up (follow-up).

**Table 1:** Intervention and data collection timeline for each cluster.

	2016						2017					
Study Year and Month	Apr-May	May-Jun	End Jun	Jul-Sept	Sep	Nov-Dec	Apr-May	May-Jun	May-Jun	Jul-Aug	Sep	Nov-Dec
Calendar Time	1		2	3	4	5	6		7	8	9	10
Cluster 1	O	X	O	O								
Cluster 2	O		O	X	O	O						
Cluster 3			O		O		O	X	O	O		
Cluster 4							O		O	X	O	O

O—Data collection

X—Delivery of intervention

Teachers completed questionnaires independently. Parents completed questionnaires during a series of home visits by an Aboriginal community researcher and a research assistant. As many parents speak English as a second, third or fourth language, the Aboriginal community researchers translated questionnaires into written Kimberley Kriol and provided verbal translation into the local Aboriginal language when necessary.

Data collection using the questionnaires unavoidably overlapped with the first and last weeks of the intervention for some students. This occurred because some teachers and parents required additional time to complete the questionnaires and the unavailability of teachers during the school holidays meant that data collection could not be extended into the school vacation. Given education stakeholders had recommended the intervention be completed over one school term (ten-week period), data collection for some students needed to occur in the first and last week of the term when the intervention had just started or was finishing. All attempts were made to obtain data from a student’s original school if they moved between clusters during the study.

### Demographics

Children’s demographic information including date of birth, sex, languages spoken at home, and Aboriginal and/or Torres Strait Islander status was collected from parents.

### Identification of children with a diagnosis of FASD

Screening for and diagnosis of FASD were not part of this study. Review of participants’ electronic Department of Health medical records with parent consent identified children with a previous diagnosis of FASD. Due to the extremely limited FASD diagnostic services available in the region, it is possible that there were more affected children in the study than just those with a previously confirmed diagnosis. Children who performed poorly on study measures were subsequently referred to the local occupational therapy team for further follow-up.

### Lesson implementation and student attendance

Lesson implementation and student attendance were recorded by teachers in the study lesson manual. Lessons were coded as implemented if teachers recorded the date next to the relevant lesson with missing lesson implementation data verified with individual teachers. Students were marked as present or absent for individual Alert Program® lessons. If these data were missing, individuals’ daily school attendance was requested from the school.

### Blinding

Blinding of teachers, parents, and researchers was not possible because of the nature of the intervention and data collection methods.

### Data analysis

All paper records were entered into Qualtrics, an online survey platform. Ten percent of all paper records and data extracted from medical records were checked for data entry errors. The mean error rate across outcomes was low (0.25%). Data were cleaned and coded in IBM SPSS version 25. Descriptive statistics were produced in SPSS to summarise participant demographics per cluster, lesson implementation and attendance information, and to inspect outcome data.

Outcome data were analyzed per the study protocol [41]. Participants were eligible for inclusion in analyses if their data were available for at least one pre- and post-intervention timepoint. No participants were excluded from analyses based on intervention exposure in accordance with intent-to-treat principles of study design. The primary outcome was change in students’ SESBI Intensity Scale scores, which was analyzed using a generalized linear mixed-effects model (GLMM) and assessed at the 5% significance level. Fixed effects included intervention, sex, age, and calendar time. To detect a possible intervention lag effect, intervention was coded as 0 (pre), 1 (post), and 2 (follow-up). Calendar time was coded 1–10 (Table 1) and was included to adjust for possible secular trends [52]. Random effects for individuals and individuals nested within clusters were also included in addition to an exchangeable correlation structure. All secondary outcomes were analyzed similarly with adjustment for multiple testing using the false discovery rate as described by Benjamini and Hochberg [53]. GLMM analyses were performed in Stata version 15.1.

SESBI and ECBI Intensity Scale scores were analyzed using the ‘meglm’ command, specifying a gamma error distribution, to account for an anticipated skewed error distribution [41]. Where mixed-effects negative binomial models were found to be non-significant, SESBI and ECBI Problem Scale and BRIEF2-SF scores were analyzed using the ‘mepoisson’ command. Over dispersion was evaluated in these analyses using mixed-effects models with negative binomial error distributions (‘menbreg’ command). Predicted means and proportions were estimated directly from the fixed and random effects.

### Sensitivity analyses

Sensitivity analyses were performed for students who attended five or more Alert Program® lessons to determine the impact of intervention exposure on all student outcomes. To determine the difference in outcomes for children with and without FASD, separate sensitivity analyses was performed by including FASD diagnosis as a covariate in the models. However, an interaction between the intervention and FASD could not be modelled given the small number of children with a confirmed FASD diagnosis. Both sensitivity analyses were assessed at the 5% significance level and included the same random and fixed effects as previously described.

## Results

### Participants

**Fig 1:**
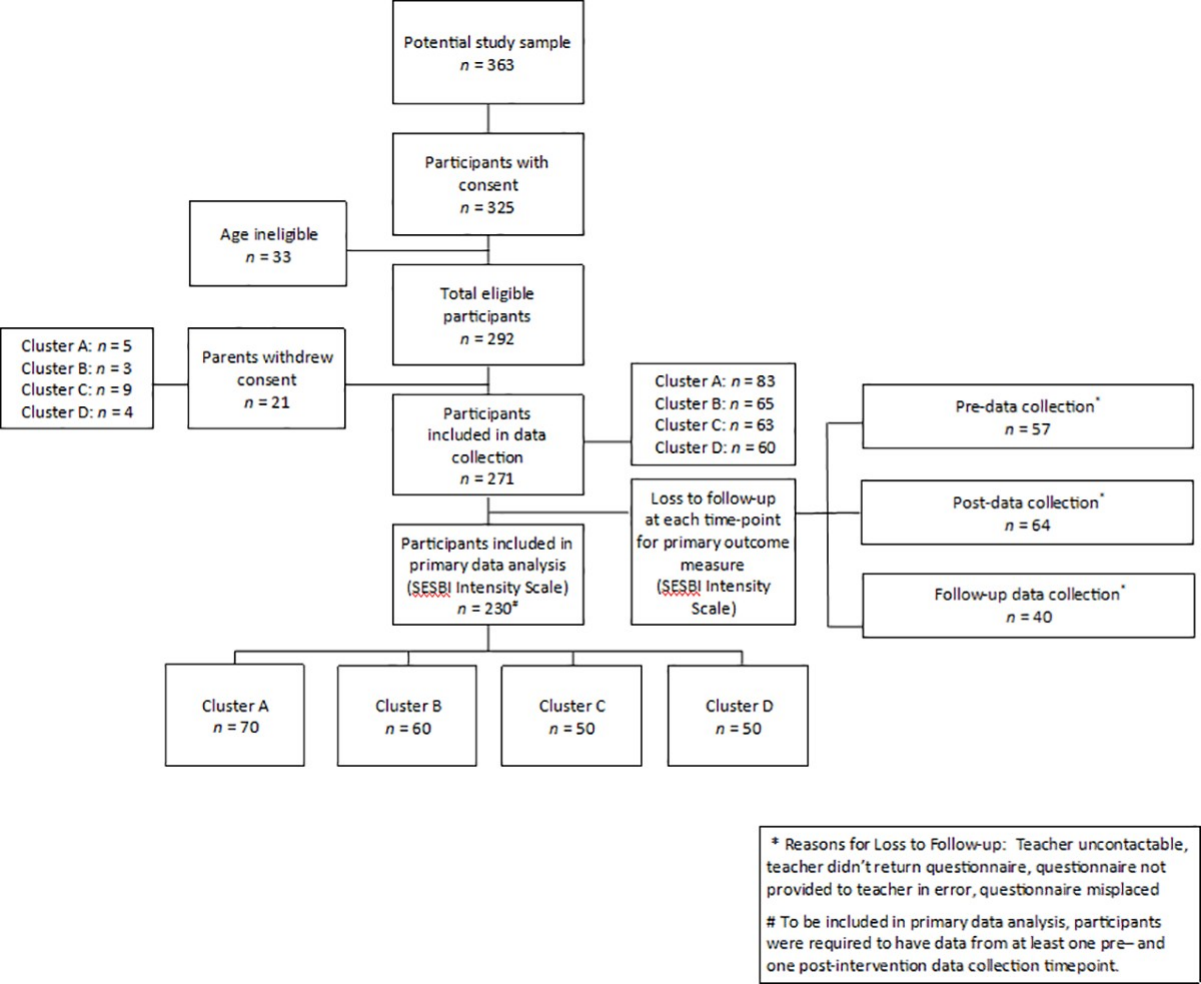
Participant flow chart.

Of the 363 students identified from 2015 school enrollment data as potentially eligible for participation in the study, 271 students (75%) participated in the research. This reduction in number was due to the fact that the actual age of children sometimes varied from school enrolment data making them ineligible (*n* = 33; Fig 1). Following initial entry of their child into the study, a further 21 parents withdrew consent as they no longer wanted to complete the battery of questionnaires. The number of participants lost to follow-up varied across data collection time-points. For the primary outcome measure, reasons included the teacher being uncontactable or not returning the questionnaires. Participants were eligible for inclusion in analyses if their data were available for at least one pre- and post-intervention time-point. Therefore, the final number of participants included in the primary data analysis (SESBI Intensity Scale) was 230. For reporting purposes, clusters were randomly assigned a new identifying code to preserve anonymity of the school clusters.

**Fig 2:**
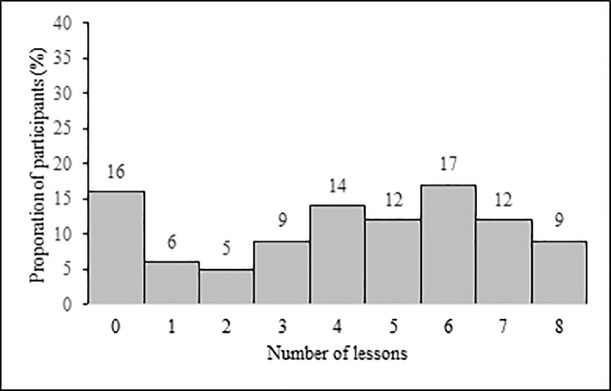
Percentage of students by total number of Alert Program® lessons attended.

Participants’ mean age at the time of consent was 8.7 (range 5.8–12.1 years). English and Kimberley Kriol were spoken by 77% and 71% of students at home respectively while 60% of students spoke two or more languages at home. Additional participant characteristics by cluster are presented in Table 2. Following parent consent, researchers accessed lesson attendance data for 264/271 students (97%) and medical records for 239/271 students (88%). The medical record review identified twelve students (5%) with a documented FASD diagnosis, a lower than expected rate. The mean number of Alert Program® lessons attended by students was 4.2 of eight possible lessons (SD 2.6). Fifty-one percent of students (135/264) attended five or more lessons (Fig 2). Lesson implementation per classroom ranged between 81 and 100%.

**Table 2:** Participant characteristics by cluster at the time of consent.

Characteristic	Cluster A	Cluster B	Cluster C	Cluster D	Total
**Number of participants**	83	65	63	60	271
**Mean age (SD)**	8.9 (1.7)	8.7 (1.8)	8.9 (1.8)	8.4 (1.5)	8.7 (1.7)
	***n* (%)**				
**Sex**					
**Male**	41 (49)	39 (60)	26 (41)	28 (47)	134 (49)
**Female**	42 (51)	26 (40)	37 (59)	32 (53)	137 (51)
**Indigenous Status**					
**Aboriginal and/or Torres Strait Islander**	73 (88)	61 (92)	60 (95)	51 (85)	245 (90)
**Not Stated**	10 (12)	<6 (<8)^a^	<6 (<8)	9 (15)	26 (10)
**Only Aboriginal languages spoken at home**	34 (41)	9 (14)	20 (32)	19 (32)	82 (30)
**School grade**					
**Grade 1**	12 (14)	14 (22)	12 (19)	13 (22)	51 (19)
**Grade 2**	10 (12)	12 (19)	9 (14)	8 (13)	39 (14)
**Grade 3**	14 (17)	9 (14)	<6 (<8)	19 (32)	46 (17)
**Grade 4**	14 (17)	8 (12)	10 (16)	6 (10)	38 (14)
**Grade 5**	21 (25)	8 (12)	18 (29)	14 (23)	61 (23)
**Grade 6**	12 (14)	14 (22)	10 (16)	0	36 (13)

^a^ Ethics committee requirements prevent reporting of results where n <6. In these instances, percentages are expressed as % = 5/cluster *n*.

#### Primary outcome

***SESBI** i**ntensity*** *Scale score*. Children’s SESBI Intensity Scale scores (*n* = 230), did not change significantly from pre- to post-intervention or pre-intervention to follow-up (Table 3).

**Table 3:** Estimates of the effects of the Alert Program® for primary and secondary outcomes as rated by teachers and parents.

Outcome measure						
Intervention time-point	Co-E	SE	95% CI		*P-value*	FDR
***Teacher-rated outcome***			**L**	**U**		
**SESBI Intensity Scale**	**Exp(b)**					
Pre- to post -	0.97	0.03	0.91	1.03	.328	N/A
Pre- to follow-up	1.11	0.07	0.99	1.25	.072	N/A
**SESBI Problem Scale**	**IRR**					
Pre- to post-	0.59	0.04	0.52	0.67	< .001	0.004
Pre- to follow-up	1.98	0.25	1.55	2.54	< .001	0.008
**T-BRIEF2-SF**	**IRR**					
Pre- to post-	0.95	0.03	0.90	1.01	0.095	0.029
Pre- to follow-up	1.08	0.06	0.97	1.20	0.172	0.033
***Parent-rated outcome***						
**ECBI Intensity Scale**	**Exp(b)**					
Pre- to post -	1.07	0.03	1.00	1.14	.037	0.029
Pre- to follow-up	0.80	0.04	0.72	0.89	< .001	0.013
**ECBI Problem Scale**	**IRR**					
Pre- to post-	1.31	0.09	1.15	1.49	< .001	0.004
Pre- to follow-up	0.32	0.04	0.24	0.42	< .001	0.008
**P-BRIEF2-SF**	**IRR**					
Pre- to post-	1.03	0.03	0.97	1.09	.326	0.042
Pre- to follow-up	0.89	0.04	0.81	0.97	.007	0.021

Co-E = exponentiated co-efficients adjusted for age, sex, calendar time, cluster and repeated measures; SE = standard error; 95% CI = confidence interval, lower limit and upper limit; P-value = probability value; FDR = false discovery rate; Exp(b) = exponentiated beta; IRR = incidence risk ratio.

#### Secondary outcomes–teacher questionnaires

Children’s SESBI Problem Scale scores (*n* = 220), significantly decreased by a factor of 0.59 from pre- to post-intervention (Table 3). However, SESBI Problem Scale scores significantly increased from pre-intervention to follow-up by a factor of 1.98. This indicates that teachers rated some students’ behavior as less of a problem post-intervention, but as more of a problem at follow-up. Both results remained significant following adjustments for multiple testing. There were no significant differences for the teacher-rated BRIEF2-SF scores (*n* = 231) from pre- to post-intervention, or pre-intervention to follow-up.

#### Secondary outcomes–parent questionnaires

Children’s ECBI Intensity (*n* = 209) and Problem Scale (*n* = 206) scores, increased significantly from pre- to post-intervention by factors of 1.07 and 1.31, respectively (Table 3). However, the Intensity scale result was no longer significant following adjustment for multiple testing. There were no significant changes in parent-rated BRIEF2-SF scores (*n* = 209) at post-intervention compared to pre-intervention. Compared to pre-intervention, at follow-up the parent-rated ECBI Intensity and Problem Scales, and parent-rated BRIEF2-SF scores, were significantly lower by factors of 0.80, 0.32 and 0.89, respectively. This demonstrates that some parents observed improvements in their children’s behavior and executive functioning at follow-up compared to before the Alert Program® intervention commenced. All three results remained significant following adjustment for multiple testing.

### Sensitivity analyses

#### Five or more Alert Program® lessons

A sensitivity analysis comparing the effect of the intervention in children who received the full intervention dose compared to those who did not could not be performed due to the small number of children who received the full intervention dose. However, a sensitivity analysis was performed comparing the effect of the intervention in children receiving five or more Alert Program® lessons compared to those who did not. These results are presented in Table 4. The results are similar to results of the primary and secondary outcome analyses (Table 3). Interestingly the one exception was parent-rated ECBI Intensity Scale scores which increased significantly from pre- to post-intervention by a factor of 1.12.

**Table 4:** Estimates of the effects of the Alert Program® for primary and secondary outcomes as rated by teachers and parents for children who attended five or more Alert Program® lessons.

Outcome measure			95% CI		
Intervention time-point	Co-E	SE	L	U	*P-value*
***Teacher-rated outcome***^***a***^					
**SESBI Intensity Scale**	**Exp(b)**				
Pre- to post-	0.96	0.04	0.88	1.05	.417
Pre- to follow-up	0.89	0.08	0.74	1.06	.184
**SESBI Problem Scale**	**IRR**				
Pre- to post-	0.57	0.05	0.47	0.68	< .001
Pre- to follow-up	1.61	0.31	1.10	2.36	.015
**T-BRIEF2-SF**	**IRR**				
Pre- to post-	0.94	0.04	0.87	1.02	.114
Pre- to follow-up	1.00	0.08	0.86	1.17	.963
***Parent-rated outcome***^***b***^					
**ECBI Intensity Score**	**Exp(b)**				
Pre- to post-	1.12	0.04	1.04	1.21	.003
Pre- to follow-up	0.75	0.05	0.65	0.86	< .001
**ECBI Problem Score**	**IRR**				
Pre- to post-	1.22	0.11	1.03	1.45	.019
Pre- to follow-up	0.40	0.08	0.28	0.59	< .001
**P-BRIEF2-SF**	**IRR**				
Pre- to post-	1.07	0.04	0.99	1.15	.076
Pre- to follow-up	0.84	0.05	0.74	0.95	.005

Co-E = exponentiated co-efficients adjusted for age, sex, calendar time, cluster and repeated measures; SE = standard error; 95% CI = confidence interval, lower limit and upper limit; P-value = probability value; FDR = false discovery rate; Exp(b) = exponentiated beta; IRR = incidence risk ratio.

^a^ SESBI Intensity Scale *n* = 132, SEBI Problem Scale *n* = 130, Teacher BRIEF2-SF *n* = 123.

^b^ ECBI Intensity Scale *n* = 124, ECBI Problem Scale *n* = 123, Parent BRIEF2-SF *n* = 124.

### FASD diagnosis

Results of sensitivity analyses performed by including FASD diagnoses as a covariate for both teacher and parent-rated outcomes were consistent with results from models that excluded FASD as a covariate (Table 3).

## Discussion

This study evaluated the effectiveness of an 8-hour teacher-delivered Alert Program®, manualized and adapted to the local context, that aimed to improve self-regulation and executive functioning skills of primary school aged students in the remote Fitzroy Valley. Effect was measured using teacher-rated and parent-rated outcomes. Importantly, the study resulted from a locally identified need for a practical, contextually relevant intervention to support children’s behavioral, cognitive, and emotional regulation. High reported rates of self-regulation impairment, exposure to ELT and alcohol prenatally, had been identified in a previous study reporting FASD prevalence.

While the results did not fully support the study hypotheses, the Alert Program® reduced the frequency of children’s disruptive behavior (as a proxy for improved self- regulation) and improved executive functioning skills, as measured by parent-rated standardized outcome measures. It is promising, that teachers rated student behaviors as significantly less of a problem immediately after the Alert Program® had finished despite reporting that the frequency of school behavior problems remained unchanged. This may reflect increased self-efficacy of teachers to manage problem behaviors, despite behaviors continuing among children. Providing information to teachers about high local FASD prevalence and rates of executive functioning impairment, coupled with training in self-regulation concepts, may have altered teacher perceptions of child behavior and executive functioning during treatment [54, 55]. Teachers may have been more understanding or tolerant of certain behaviors or felt better equipped to manage problem behaviors throughout the intervention period. However, these results were not maintained at follow-up. This could indicate that to sustain improvement, teachers require additional training or coaching to continue embedding Alert Program® language and strategies throughout the school day after completion of formalized lessons.

The use of ecological measures (SEBSI, ECBI and BRIEF2-SF) may partly explain the variability of results between teacher and parent ratings of student behavior and executive functioning. While ecological measures can assess the everyday meaning and presentation of behavioral problems specific to real-world contexts such as the classroom and home [56, 57], the objective measurement of these can be influenced by the raters’ own ideals, beliefs, and attitudes [57]. Many of these beliefs are influenced by differences between Aboriginal and Western cultural norms [58, 59] and because of this teachers and parents may over- or underestimate poor child behavior compared to each other. Factors such as self-efficacy of caregiving skills or the perception of ones’ teaching or parenting abilities being scrutinized, may have influenced ratings. This is especially pertinent given the absence of ecological executive functioning and self-regulation assessments normed for use with Australian Aboriginal children [58, 60].

Varying student attendance may have impacted results given the strong association between behavioral problems and low school attendance reported in the literature [61, 62]. Only 50% of student study participants received more than half of the Alert Program® lessons; a much lower rate than other Alert Program® studies that have reported intervention exposure [33, 37]. The small sample of participants who received exposure to the full intervention dose meant sensitivity analyses could not be performed for this group. However, sensitivity analyses performed for children who received five or more Alert Program® lessons revealed similar findings for teacher and parent-rated outcomes as those emerging from the primary and secondary outcome analyses.

It is possible therefore that children who stood to benefit the most from the program were the students who received the least exposure given their absence from school. This is one possible explanation for why significant changes were not detected via teacher-rated outcome measures. School attendance in the Kimberley region, which encompasses the Fitzroy Valley, is typically the lowest of any Western Australian school district [63]. Reasons for this include family travel to surrounding communities to attend funerals, medical appointments, and cultural events. The multiple biopsychosocial influences on child behavior and development in a remote community setting may require multilayered interventions. It is possible that a single intervention while necessary, was not sufficient to demonstrate a measurable effect. Any future efforts to identify or design contextually appropriate multi-model interventions must be done so in partnership with local communities and their stakeholders.

One real-world, positive outcome from this study was the provision of extra teacher support and training as requested by communities and schools during the Lililwan FASD prevalence study. School staff received support to manage difficult classroom behaviors [25] through training in self-regulation and Alert Program® concepts. To ensure cultural congruence the training, intervention, and resources were designed in partnership with local occupational therapists, then piloted at a local school, and further refined based on local teacher feedback prior to this study [30]. The whole-of-school and region approach to Alert Program® teacher training and classroom delivery meant children could receive immediate access to strengths-based classroom strategies and accommodations which was identified as a community priority [64].

Another positive outcome of study implementation was community acceptability of the study, and responsiveness to expressed community needs. Study acceptability was made possible through the existing relationships many research team members had established with, or as members of, the Fitzroy Valley Marulu FASD/ELT Strategy leadership group. Several researchers also had enduring relationships with Aboriginal community leaders, health, and education stakeholders through their previous or current roles working for local Aboriginal organisations, schools, and health agencies. These relationships enabled researchers to partner with key stakeholders, including the local Aboriginal cultural health service, allied health staff, and schools, to develop and refine the intervention, outcome measures, and other study protocols [30].

Although findings were mixed, a highly valuable project outcome was the employment and training of 18 local Aboriginal community researchers from various Fitzroy Valley communities to provide invaluable expertise in cultural protocols, language translation, relationship facilitation between researchers and families, and data collection. A separate capacity building grant also enabled several community researchers to complete a nationally recognised Certificate II in Community Services to support employment opportunities following the study. Non-Aboriginal researchers also received vital cultural guidance and training from community researchers, Aboriginal organisations, and Elders to better understand Indigenous ways of knowing and doing, and to help them work in a culturally secure way. Without the local knowledge and cultural guidance of the community researchers or local stakeholders, it is unlikely the project would have been feasible given the challenges of conducting multi-site research in remote community schools.

### Limitations

The challenges of intervention research in remote communities are many and varied, even when an experienced research team that includes many Aboriginal community researchers puts measures in place to address these potential limitations. The findings of the current study therefore, must be interpreted in the context of several important limitations. As described elsewhere [41], the stepped-wedge cluster randomized control trial design originally proposed was not feasible in the remote community context. This necessitated a non-standard study design and meant that sample size calculations could only be estimated conservatively. While it is possible the study was underpowered for some outcomes, the largest possible number of participants were recruited from the entire available study population. A repeated measures design was employed to increase the likelihood of detecting any significant effects of the intervention on student outcomes. Surprisingly, despite the very high reported rates of FASD in the Fitzroy Valley, and substantial efforts to obtain information and refer children for diagnosis, only 12 students were identified with a confirmed FASD diagnosis during the Department of Health medical record review. This impacted the study’s ability to detect an interaction between diagnosis of FASD and impact of the intervention. Screening for prenatal alcohol exposure and subsequent FASD diagnosis as part of this study was not feasible due to budget constraints and the limited local diagnostic capacity. Given FASD prevalence in the Fitzroy Valley was established as 194 per 1000 children [26], it is likely that many children in the current study had undiagnosed FASD. Should this be the case, future efforts to identify effective interventions for children in regions such as the Fitzroy Valley may be limited.

Blinding of teachers, parents, and researchers to the intervention was not possible given the nature of the intervention and how the data needed to be collected. It is recognised that outcomes of non-blinded studies may be biased with a risk of inflating the likelihood of a type I error. Unblinded teachers and parents may have taken greater note of target behaviors at the post and follow-up data collection time-points, which could have affected the results.

In some instances, different teachers and parents completed questionnaires for the same student across time-points for reasons that included teacher absences or resignations, and parent availability. This may have impacted the results. The frequency of respondents changing was not recorded and is a limitation of this study.

There was an unavoidable overlap between the first and last Alert Program® lessons and pre- and post-intervention data collection, to enable sufficient time for teacher and parent questionnaires to be administered within the school term. Therefore, some students may have received exposure to the Alert Program® prior to completing the pre-intervention outcome measures, or may not have received the full intervention dose prior to completing the post-intervention outcome measures. Implementation fidelity of lesson activities is unknown because some parents declined consent for researchers to film lessons. A random audit using film was not possible.

## Conclusions

While this study demonstrated no effect of the 8-week Alert Program® intervention on children’s disruptive behaviours as measured by the primary outcome, it provides important information for future research evaluating urgently needed self-regulation interventions for Aboriginal Australian children living in remote communities. The lack of effect demonstrated in this study must be viewed cautiously and may be due to many factors. These include that the program as delivered is ineffective, but also that the limitations of the study meant that we failed to find an effect when one existed. This may be due to the complex setting in which this work was conducted including the lack of validated measures available for use in Aboriginal populations.

Future research is urgently needed to identify, in consultation with local communities, effective self-regulation programs for supporting teachers, students, and their families to manage problematic behaviours that prevent students from achieving success at school. Despite mixed findings, this study was an important first step in adapting and evaluating one available self-regulation program for use in remote Australian Aboriginal community schools where access to evidence based interventions is extremely limited.
