# Correction: School-based intervention to address self-regulation and executive functioning in children attending primary schools in remote Australian Aboriginal communities

**DOI:** 10.1371/journal.pone.0236485

**Published:** 2020-07-22

**Authors:** PLOS ONE Staff

## Notice of republication

This article was republished on July 6, 2020, to remove a paragraph that was incorrectly included in the originally published article. The publisher apologizes for the errors. Please download this article again to view the correct version.
